# Educator perceptions on the benefits and challenges of loose parts play in the outdoor environments of childcare centres

**DOI:** 10.3934/publichealth.2019.4.461

**Published:** 2019-10-29

**Authors:** Rebecca A Spencer, Nila Joshi, Karina Branje, Jessie-Lee D. McIsaac, Jane Cawley, Laurene Rehman, Sara FL Kirk, Michelle Stone

**Affiliations:** 1 School of Health & Human Performance, Dalhousie University, Halifax, Canada; 2 Faculty of Education and Department of Child and Youth Study, Mount Saint Vincent University, Halifax, Canada; 3 Healthy Populations Institute, Dalhousie University, Halifax, Canada

**Keywords:** early childhood, education, loose parts, outdoor play, educator perspectives

## Abstract

It is important to consider physical activity and movement in early life to ensure children establish and maintain healthy physical activity patterns. Recent evidence has highlighted the importance of outdoor play and the childcare environment. Active outdoor play, especially free play, supports independence, self-regulation and allows children to explore their world and make decisions. Loose parts or open-ended materials are natural or synthetic resources that can be used in more than one way, allowing children to experiment through play. Incorporating loose parts into play environments creates opportunity for new play experiences. Despite growing evidence supporting loose parts play, the perspectives of childcare providers on the benefits and challenges of this type of play have been overlooked. The purpose of this study was to identify the benefits and challenges of incorporating loose parts play into the outdoor environments of childcare centres, from the perspectives of educators who took part in the Physical Literacy in the Early Years (PLEY) project. PLEY is a larger, mixed methods intervention study with the goal of evaluating a loose parts intervention in early childcare settings. This portion of the project used qualitative description to explore educators' perspectives. Data were collected using focus groups (n = 15) with early childhood educators (n = 3–5 in each group). Thematic analysis was used to identify five themes relating to benefits, and four themes relating to challenges. Benefit themes included: loose parts enable children to take risks; loose parts spark creativity and imagination; loose parts contribute to problem-solving abilities; loose parts cultivate independence and confidence; and loose parts build relationships and leadership. Challenges included: apprehension of loose parts; loose parts as a novelty; sustainability of loose parts; and loose parts present challenges with storage. Overall, we found educators perceived outdoor loose parts play to have multiple social and cognitive benefits for preschool-aged children that are critical for optimal growth and development, and overall health and wellness.

## 1. Introduction

Several reviews have documented demographic, biological, environmental, social and psychological influences of physical activity in early life [10],[11], with multiple studies highlighting the significance of outdoor play, and also noting the role of the childcare environment and childcare providers [12]. Children are more active when they play outdoors [13],[14]. Approximately 60% of Canadian preschoolers attend some form of child care [15], making this an ideal setting to support outdoor play and physical activity in early life [16]. With mounting evidence citing the significance of outdoor play in early life to children's physical and mental health, and cognitive and social development [17], and advocacy for children's right to play outdoors and take risks in outdoor play [14], there is interest in exploring the potential of the childcare environment in supporting children's outdoor play and increasing physical activity. Importantly, the literature indicates positive associations between children's time spent outdoors in childcare and physical activity behaviour [11],[18], highlighting the contribution of childcare outdoor play to children's physical activity behaviour [19].

While play is universally understood as critical for multiple aspects of child development, structured play is often guided by adults [20]–[22]. Because free play permits internally-motivated freedom of choice, and activity that is self-directed and open-ended [23], it also supports the development of independence, and emotional and behavioural self-regulation. Furthermore, free play allows children to create their own activities, explore their social world and make their own decisions [24],[25]. Active outdoor play offers children more opportunity for free play, and affordances to enhance not only their physical activity, but other aspects of growth and development [13],[26]. One way to encourage active outdoor play is to incorporate loose parts into the play environment as these create the opportunity for creative, dramatic, exploratory, cooperative or constructive play [22].

Loose parts or open-ended materials are natural or synthetic resources that can be used in more than one way, thereby allowing children to experiment through play [27]. By incorporating loose parts into children's play spaces and giving them little or no instruction, children are provided with the opportunity to engage with objects how they choose [24]. Loose parts play allows children to create their play experiences based on their ideas and goals rather than materials with one purpose predetermining their play [28]. Loose parts can encourage children to explore their environments, take risks during play, and develop confidence and motivation [29]. Several studies have explored the benefits of outdoor loose parts play within early years settings, finding a wide range of cognitive and socio-emotional benefits, including happiness at school, social benefits and enhanced exploratory, creative and dramatic play [22],[24],[30]–[34]. Others have noted loose parts have potential to promote physical development as well, through the encouragement of active play and development of physical literacy and fundamental movement skills [22],[24],[29]. Importantly, others have noted children preferred loose parts play than play with structured toys [32].

Despite growing interest in exploring the benefits of outdoor loose parts play, there are gaps in the existing evidence. While there is attention to the benefits of loose parts play for children's health, further research is needed to explore the socio-emotional domain, as well as educators' perspectives [24],[30]. There is little understanding in the scientific literature of the challenges of incorporating loose parts into childcare outdoor environments as a way to enhance children's health, and the value of doing so, from the perspective of childcare providers or educators. As children spend a majority of their time in educational settings, and these settings have been identified as important for play, development, and physical activity intervention, it is critical that we better understand the perspectives of educators. Educator perspectives could identify the unique benefits of outdoor loose parts play to children's development, which could provide further support for their value in optimizing children's health and wellness.

Educators believe that when children move outdoors it promotes learning [35], and more specifically, it facilitates the development of confidence and competence [36]. As educators influence the way the children interact with the play environment [36] it is critical to involve them when attempting to improve early childhood educational programs [35]. Educator perspectives could also identify ways in which childcare providers integrated loose parts into childcare environment outdoor spaces, the challenges and barriers they encountered, and how they overcame these to support and maintain this type of unstructured, child-directed, free play. These insights could in turn help other educators become more comfortable in integrating outdoor loose parts play into their spaces as a means of facilitating hands-on exploration that meets children's natural sense of inquiry and discovery. Ultimately, these perspectives could support more widespread adoption of outdoor loose parts play in child care centres on a local, national and even international level, a shift in practice that would benefit children's physical, cognitive, social and emotional health, and the health and wellbeing of communities. The purpose of this study is therefore to identify the benefits and challenges of incorporating loose parts play into the outdoor environments of childcare centres, from the perspectives of educators who took part in the Physical Literacy in the Early Years (PLEY) project, as a way of promoting, supporting, and sustaining outdoor loose parts play in childcare settings in Nova Scotia (Canada).

## 2. Materials and methods

### 2.1. Study design

This study represents a qualitative component of a larger, mixed methods intervention study. The protocol for the larger study, the Physical Literacy in the Early Years (PLEY) project has been previously described ([37]; ID# ISRCTN14058106). The overall goal of the PLEY project was to evaluate the use of a loose parts intervention in comparison to standard outdoor play practice in early childcare settings. The intervention also sought to improve educators' attitudes and beliefs around incorporating loose parts into outdoor play. The study was reviewed by the university's institutional review board, and written consent was achieved for all participants. The broader PLEY study took place in a total of 19 childcare centres (11 intervention). Sixteen sites (8 intervention) were originally recruited, with three additional intervention sites added as s second cohort a year later. Sites were spread across Nova Scotia, across a spectrum of rural to urban settings and representing a variety of socioeconomic positions. Ten of the sites operated as non-profits, while nine operated commercially. All sites (n = 19) served toddler and preschool-aged children while many also served infants (n = 15), and some also served school-aged populations (n = 9). Total enrolment for the included childcare centres ranged from 36 to 162 children, with mean enrolment of 82 children.

In addition to quantitative study components, qualitative data were collected to richly explore the perceptions of educators regarding the intervention in more depth. Given the exploratory nature of this component of the project, Qualitative Description (QD) was used. QD is a qualitative method or approach that focuses on participant perspectives and describing lived experiences [38],[39]. QD, while less interpretive than other qualitative methodologies, is appropriate for understudied areas and permits rich description of participant perspective, emphasizing their voice [38],[39]. In this study, participant perspectives specifically relate to the benefits and challenges of incorporating loose parts play within childcare settings. Benefits relating to physical activity, physical literacy and fundamental movement skills of children were not the focus of this analysis.

### 2.2. Intervention

The PLEY project intervention involved integrating loose parts into outdoor play spaces within 19 childcare centres (11 intervention) across Nova Scotia for a period of between six and eight months. Intervention sites were provided with a loose parts kit, which included buckets and lids (variety of shapes and sizes), rope and pulley, tree cookies, milk crates, a package of hose tube, 20+ balls (a variety of sizes and weights), wood pieces, a bread tray, large cardboard tubes, funnels of different sizes, a tarp, 5′ planks, 5′PVC tubing (4” and 2” diameter), rocks and tires [37]. Control sites were provided with loose parts kits at the end of the study. The project employed the socioecological model to understand and address multiple levels of influence, and the RE-AIM framework is being used to evaluate the reach, effectiveness, adoption, implementation, and maintenance of the intervention [40],[41]. Site recruitment procedures and other details about the intervention and other outcome measures are provided elsewhere [37].

### 2.3. Data collection

Data were collected using focus groups with early childhood educators. Purposive sampling was used to recruit participants who would be familiar with the PLEY intervention [42]. As such, all educators from intervention centres were invited to take part in the focus groups. Focus groups occurred at 3-months and 6-months following the intervention. A total of 15 focus groups took place, nine at three-months, and six at 6-months, with 3–5 participants in each group, which is consistent with qualitative description methodology [38],[39]. This approach also allowed us to gain insight on educator experiences throughout the intervention period. Focus groups included educators from a variety of intervention sites and took place at the childcare centres or at a public location. All intervention sites were represented in focus groups, which each lasted approximately 45–60 minutes and involved questions regarding active outdoor play, loose parts, risk-taking, and the intervention (please see Appendix A for the semi-structured focus group guide). Focus groups were facilitated by a combination of students and members of the research team. Focus group facilitators were trained together by project coordinators, and included a diverse group, representative of a variety of ages and backgrounds, all of whom identified as women. Some focus group facilitators had relationships with participants that would have developed through this intervention study. All focus groups involved a facilitator and a note-taker.

### 2.4. Data analysis

All focus group and interview data were audio-recorded and transcribed verbatim. Data were organized using Microsoft Word (version 16.16.3) and imported into QSR NVivo 11 for analysis. Data analysis was conducted primarily by research staff and guided by a senior member of the research team. Four project coordinators and research assistants began by reviewing the same transcripts using open inductive coding, guided by Miles and Huberman, and Braun and Clarke [43],[44]. Frequent meetings were held to discuss codes and a codebook was developed collaboratively and iteratively. To develop consistency, two coders coded each transcript early in the analysis, allowing exploration and discussion of similarities and differences. Once consistent coding and a codebook were established, the remaining transcripts were coded using a single coder. Thematic analysis was guided by Miles and Huberman (1994) and Braun and Clarke (2006), using a collaborative process by which relationships between codes and trends in the data were identified and discussed. Final presentation of themes was agreed upon by the group. Due to the nature and timeline of the work, we were unable to review the results with the participants. This collaborative process, peer review, and field notes contribute to the dependability, authenticity, credibility, and transferability of the results [45],[46].

## 3. Results

From the focus group data, five themes relating to benefits of loose parts play, as perceived by educators, were developed. These were: loose parts enable children to take risks; loose parts spark creativity and imagination; loose parts contribute to determination and resilience; loose parts cultivate independence and confidence; and loose parts build relationships and leadership. Additionally, four themes relating to challenges were identified: apprehension of loose parts; loose parts as a novelty; sustainability of loose parts; and loose parts present challenges with storage. Below, themes are supported by quotations, which are identified using focus group numbers that took place at 3-months (3M) or 6-months (6M) and with the original cohort (OC) or new cohort (NC).

### 3.1. Benefits of loose parts play

**Loose parts enable children to take risks.** This theme relates to how loose parts were perceived as contributing to the development of a healthy conceptualization of risk taking and risky play, for both adults and children. Educators discussed how the use of loose parts seemed to help children become less fearful of active outdoor play and learn to take progressively more healthy risk. One educator said, for example, *“Even walking on the balance beam […] like they add the risk to it. So ‘o.k. I've done this balance beam enough times, I'm going to add something to it. So maybe I'm jumping from that to the spool, into the hoola hoop’ […] and they just keep extending on to it to keep it more of a little risk”* (3M NC Group 3). Another connected this progress to loose parts, saying, *“they were more eager to take risks […] after using these materials in different ways, they were more eager”* (3M NC Group 4). Educators also discussed their perception that loose parts play helped children to better understand their capabilities and capacities. One said, *“when they fall […] they'll just get up and go”* (3M NC Group 1), and another described an example, saying, *“she'll […] lose her balance, and usually that's something that she would never go back to […] but every day she goes back and she tries […] it's really nice to see that she keeps going back even though she knows that she could get hurt”* (3M NC Group 2), demonstrating a child's development of healthy risk taking.

This theme also extended to the educators' perceptions of risk, where they discussed their own progressive learning and acceptance of healthy risk over time. One said, for example, *“You're just so used to saying oh be careful, be careful, be careful, don't do that”* (3M NC Group 1), while another described a shift in this mentality, saying, *“before I would probably be more scared but now that we have all the different parts […] we're taking more risks”* (3M NC Group 1). Educators discussed how *“through hearing about it and learning about it, you feel more confident in yourself to let your children take the risks”* (3M NC Group 1), how loose parts play is *“taking me out of my comfort zone”* (3M NC Group 1), and *“changes my idea, definitely expanding what they can and cannot do”* (3M NC Group 3). One educator described her experience of this shift in perception over time, saying, *“when they started doing it I was like ‘yah let's not do that, why don't you get down?’ but then they kept doing it because they really love it […] and then at first I was spotting them and then I realized they don't need me, I'm just going to sit back and keep my eye on them, and now I just let them go, so it's just a build-up over time and me realizing that they really don't need me right there”* (3M NC Group 3).

**Loose parts spark creativity and imagination.** Another benefit of loose parts play related to how loose parts inspire imaginative and creative play. This was frequently related by the participants to the open-ended nature of loose parts play. One said, *“there's so much they could do with it”* (3M NC Group 1), while another said, *“ways they can use it that we never would have imagined”* (3M NC Group 1). One educator described this as a benefit for development, saying, *“it's just kind of the nature of loose parts, where they're […] so open-ended and you can do anything with them that it does like nurture every area of development, because like you know there's that freedom and that exploration”* (3M NC Group 1). Others connected this benefit to boredom, saying, *“they're spending longer time with the activities rather than moving from activity to activity to activity”* (6M NC Group 2), while another described an associated shift in perspective: *“we kind of thought like oh this is going to be really new and exciting and then you know probably after a couple of weeks […] they'll be bored of it again, but every day they come up with something new because it can be used in so many different ways […] they haven't gotten bored”* (3M NC Group 1).

Others discussed the *“many possibilities”*, saying loose parts *“sparked their creativity”*, and how *“the more I give them space, the more their imagination flourishes”* (3M NC Group 4). Several educators described the many imaginative and creative scenarios used in loose parts play. One said, *“they take adventures every day”* (3M NC Group 4), while another described a situation in which children were using the loose parts to re-enact scenes from a movie, saying, *“he just enhanced it […] so they're climbing up on the climbers and everything to enhance the role play”* (3M NC Group 1). Another described a pretend camping trip, saying, *“like the tarp they make on the deck, they make like a little tent out of it and camp out, and then they use the wood to make a fire to roast marshmallows”* (3M NC Group 1). This imaginative and creative play was stimulated by and enhanced by the loose parts, which seemed to spark a variety of ideas and possibilities for active play.

**Loose parts contribute to determination and resilience.** Another theme relating to the benefits of loose parts play was how they contribute to aspects of problem-solving, determination, and resilience. Educators discussed this theme, connecting loose parts to cognitive development, planning and goal setting, and resilience and perseverance. Participants discussed many examples of children *“trying to figure it out”* (6M OC Group 3), *“the cause and effect and the trial and error”* (6M OC Group 3), and how with loose parts, *“they're more determined to see it through to the end than if they were in the classroom”* (3M NC Group 2), or how *“they can think of how they're going to make it work, they have a plan”* (6M OC Group 4). One described watching a child, saying, *“you could really see the wheels turning, and their problem-solving through the whole thing, really thinking outside the box”* (3M NC Group 4). Similarly, an educator described a child's *“determination to get their foot over the tire, to hold themselves up with the rope, to touch the tree and hold the tree”* (3M NC Group 1). A participant also described how, *“they weren't getting frustrated […] they just kind of kept at it and invited other children to come over and try”* (3M OC Group 1), while another discussed how beneficial this is, saying, *“it's amazing to watch them especially when they figure it out, like there's no greater joy than seeing a kid when they just struggled […] and then they have this aha moment”* (3M NC Group 1).

**Loose parts cultivate independence and confidence.** The participants discussed how loose parts promote independence and confidence building. Many discussed how loose parts contributed to a growth in confidence, self-esteem, and pride. One said, for example, *“it ties into self-esteem yah it's like that sense of pride and accomplishment from when they are able to successfully do whatever it is, they want to do”* (3M OC Group 1), while another said, *“it also builds their confidence because they have a plan, they're going to execute the plan and they're like, ‘I can do it’. So I definitely feel like their confidence has boosted”* (3M NC Group 1). Others discussed examples of shy or introverted children finding opportunities as well. They discussed children who *“moved on from nervous to not cautious at all”* (6M OC Group 2) and how loose parts *“gave an opportunity for the children who weren't always so physical a way to do that […] to find their own comfort zone”* (3M OC Group 5). An educator described a particular child by saying, *“he's generally a more quiet kid […] you could see like the pride in his invention, as all the kids lined up […] and he was like ‘I made that’ and it was really cool, he was so confident in himself”* (3M NC Group 3). Finally, as part of this theme, several participants discussed how loose parts helped children develop independence, and how they, as a result, needed less direction. Many participants discussed the benefit of *“independent thinking”* (3M NC Group 4) and how educators perceived that *“we don't have to help them do it, they can do it on their own”* (3M NC Group 4).

**Loose parts build relationships and leadership.** A final benefit associated with loose parts play was related to social benefits of leadership, relationship building, inclusion, and reduced conflicts. Most participants discussed the social benefits of loose parts play encouraging children to work together and cooperate on tasks. One said, for example *“I found a lot more cooperative play and less fighting when we brought the loose parts in cause like all of a sudden, they were you know working together to try to figure out the pulley or working together to make things and build things”* (3M OC Group 1). Others commented on how children *“would kind of partner up and work together”* 6M NC Group 2), and how they were *“cheering each other on and they were excited […] just encouraging more kids to come do it with them”* (3M NC Group 1). An educator described, for example, how *“one of the little boys […] he thought. I'm going to put this plank here’ and so then he started walking on it, and children saw him doing it and they were like, ‘oh I want to do that too’ […] and then it just got bigger and bigger […] and they all kind of worked together to add to it”* (6M NC Group 2).

Others discussed the social benefits in relation to leadership and inclusion. Several participants discussed how loose parts facilitated new friendship opportunities and highlighted a mentoring role for older children. One said, for example, *“it started some new friendships that other kids, maybe even some older kids, saw like hey he's more capable of doing, you know, bigger kid things”* (3M NC Group 2), while another said, *“it was really nice to see how they worked together and were mentoring each other and cooperating and helping the younger ones”* (6M NC Group 1). Finally, some participants discussed how loose parts can contribute to reduced conflict. Several noted how the cooperative nature of loose parts play resulted in better sharing, and how children *“didn't argue, nobody grabbed”* (3M NC Group 4). One described their perception that *“because they've built it together, they don't try to, you know, push each other off or jump in front of each other as much”* (3M NC Group 3). Others noted *“a lot less conflict”* (6M NC Group 1), saying, *“they're burning off their energy, that they're coming in calmer, more focused”* (3M NC Group 1), suggesting the physical activity benefits of loose parts play connect to social emotional learning as well.

### 3.2. Challenges of loose parts play

**Apprehension of loose parts.** One of the potential challenges associated with loose parts play is the apprehension associated with loose parts, especially when they are first introduced. This apprehension was described with reference to the children as well as from the adult perspective (parents and educators). An educator described, for example, how *“some of our children seemed lost at first […] for some they just stood at the back and waited […] until a recognizable activity had started before going anywhere near”* (3M NC Group 1). Others noted how loose parts can *“take a little while for them to adjust”* (3M OC Group 1), referring to the children, and also how they might be perceived as, *“a little intimidating at first for children and for educators sometimes if they're not used to that”* (3M OC Group 1), noting the potential apprehension for educators as well. Another educator described potential parental apprehension, saying, *“I also have to educate the parents as well. This is what we're doing, this is why we're doing it, […] parents really didn't understand […] you know why this is happening, why is there, you know, it looks like garbage to them on the playground right, so we try to educate them a lot and explain how important it is for outside play”* (3M NC Group 3).

**Loose parts as a novelty.** Sometimes, participants discussed the potential for loose parts to be perceived as successful only due to their novelty. An educator said, for example, *“it was exciting to see them and you know just putting new, different size boards or things like that […] I didn't see like, I didn't really observe a huge change other than excitement at first for just something different”* (3M NC Group 1), noting that the benefits of loose parts may be due to the excitement around having something different. Others discussed how they feel like they are *“trying to keep the children engaged and keep them thinking and trying, push them to be creative with different little projects”* (3M OC Group 5). Finally, one educator noted that the benefits of loose parts may not be able to extend outside of their childcare environment *“if these kids go home and they don't have those same opportunities to go outside into their backyard and play with loose parts”* (6M OC Group 2), noting a potential *“disconnect”* (6M OC Group 2).

**Sustainability of loose parts.** Occasionally, educators discussed the sustainability of the loose parts intervention, in that they either wanted more, or more durable parts. Simply put, one educator said, *“we need more stuff”* (3M NC Group 2), while another noted there were *“not enough materials for the amount of children”* (3M NC Group 2). Others noted that *“there were some things that did break”* (3M NC Group 3). Finally, one educator said, *“the main challenge is just, you know, durability of some of the materials”* (3M NC Group 4). These quotes demonstrate the challenges associated with durability of loose parts and having sufficient quantity for the children to be able to engage in loose parts play.

**Loose parts present challenges with storage.** The most consistent challenge identified with loose parts related to storage. One educator said, for example, *“storage was really hard […] we have a little shed and that's where like all the cars and all that stuff is and there's barely room for that so we have like milk crates, we have PVC pipes and the buckets are kind of like stacked as well […] that was definitely a struggle”* (6M OC Group 3), noting how storage can be a typical challenge for outdoor toys and how loose parts can add to that challenge. This was also a challenge in consideration of the Canadian climate, which often comprises cold, snowy, winters. One educator said, for example, *“we are talking a lot about storage because now that we're into the winter months if it wasn't properly put away it's frozen to the ground […] so we're kind of missing some of those favorite pieces […] so we were talking a lot about how do we have storage in our playground but yet still make the storage look beautiful and aesthetically pleasing rather than having just the material scattered throughout the playground”* (6M OC Group 3), discussing this challenge both in relation to whether, and in relation to aesthetics. Others discussed how loose parts *“get snow-covered”* (3M OC Group 4) or *“frozen in”* (3M OC Group 4) and how *“one challenge is keeping them all together”* (3M OC Group 4), reinforcing the need for proper storage of loose parts.

## 4. Discussion

This study assessed the benefits and challenges of incorporating loose parts play into the outdoor environments of childcare centres, from the perspectives of educators who took part in the Physical Literacy in the Early Years (PLEY) project [37]. This project addresses a gap in the literature by including the perceptions of educators on outdoor loose parts play in an early child care environment, which is important as educators are a critical part of the success of school-based interventions. Through qualitative description, we identified five perceived benefits and four perceived challenges as described by early childhood educators across sites that received loose parts kits as part of a larger intervention. The benefits described by educators focused exclusively on socio-emotional aspects of play—the healthy conceptualization of risk-taking and risky play, and the ways in which loose parts play sparked curiosity and imagination, or encouraged independence and confidence, while also fostering cooperative play and leadership skills among the children. By contrast, two out of the four challenges focused on practicalities of using loose parts, such as having space to store them or ways to replace or sustain a supply of loose parts. The two other challenges—apprehension about using loose parts and loose parts as a novelty—suggest broader concerns about the practicalities of their use that could potentially be overcome through continued exposure to this type of play.

Consistent throughout discussions with educators, was how the integration of loose parts in the outdoor play space has seemed to increase children's participation in risky play, as well as the educator's attitude and comfort level toward this type of play. This is consistent with previous literature, which indicates that, with loose parts, children are given the opportunity to understand their capacity for risk taking; these materials allow children to discover, and overcome uncertainty, as they adapt to the novelty loose parts add to their play, which allows them to test their limits [47]. As such, these experiences allow children to gain confidence and self-awareness, which contributes to their development of healthy risk taking [48]. This study adds further depth to existing literature to suggest that the presence of loose parts in the outdoor play space, along with supervising this type of play, may contribute to educators' understanding on how these materials enhance children's play experiences and overall health. These identified benefits show how integrating these materials within childcare centres could help educators learn that risky play contributes to different facets of child development including physical, cognitive, and social-emotional development [17],[48]. While comparing timepoints is beyond the scope of qualitative analysis, it is notable that educators seemed to become more comfortable with the idea of risky play over time.

Throughout the focus groups, educators expressed how integrating loose parts within the outdoor play space increased creativity and imagination, while helping to cultivate resiliency, determination and problem-solving skills. This is also aligned with current evidence on loose parts play which describes how the open-endedness of these materials promotes creativity, imagination and problem-solving and adds to children's cognitive development [24]. Educator's perceptions on how loose parts play promotes components of cognitive development, however, it is an emerging area for research. Our study adds valuable insight to this field, by suggesting educators were able to connect components of cognitive development such as planning, problem-solving, communication, imagination, and decision making to loose parts play. This suggests that educators not only understand the benefits of loose parts play on children's health, but the value it adds to cognitive development. This type of play allows educators to draw connections between unstructured play and cognitive development, and how components such as creativity and problem-solving are enhanced through outdoor loose parts play [24]. Further, it illustrates how learning can take place outside of the classroom and how outdoor loose parts play can provide an enriching environment for children to learn [24].

Finally, educators expressed how the integration of loose parts helped children to build relationships, develop leadership skills, and gain confidence and independence. Our research adds to the current evidence by suggesting that children using loose parts exhibit more prosocial behaviours including sharing, altruism, and inclusion. It appears as though the flexible nature of loose parts allow children to be more inclusive and aware of their surroundings. Previous research indicates that having a variety of open-ended materials allows children to be immersed in their play and allows children to focus, which may explain why educators in our study saw a decrease in conflicts and an increase in cooperative play [49]. Further, having an engaging environment with loose parts allows children to explore, learn, and create, which allows children to be physically active and take ownership of their environment [24]. Together, this may help children develop confidence in themselves and a sense of independence. Engaging in this type of play, especially in a natural outdoor play environment, allows children to develop resilience, and self-regulation which is critical for coping with stress later in life [14].

A few challenges regarding loose part play were identified by educators, which help to inform intervention strategies to support sustainable use of loose parts. They discussed how the introduction of these materials sometimes led to children feeling overwhelmed or intimidated. Likewise, once these materials were introduced, educators were concerned the novelty might wear off resulting in decreased engagement with the materials. Strategies that can be used to address the apprehension and novelty of loose parts materials would be to introduce a select variety of materials over time [29]. Other challenges noted by our educators, were the practicalities of loose parts, specifically relating to sustainability and storage. A strategy that can be used to address these challenges would be to develop partnerships with community organizations [29]. Some educators commented on how they had requested donations of both loose parts and storage materials from local businesses, demonstrating how they have taken ownership of this type of play by trying to find a way to sustain loose parts. Further, these relationships provide an opportunity to spread awareness and promote loose parts play within in communities by exposing others to the concept of loose parts. By connecting with private and public organizations like municipal recreation planners and private industry partners, and explaining how these materials contribute to children's health and wellness, more people may be informed on this type of play and its benefits, which may also help mitigate parental and educator apprehension.

Through this project, we were able to explore early childhood educators' perceived benefits and challenges of outdoor loose parts play. Educators seem to understand and appreciate the flexible nature of loose parts and the value they add to children's cognitive and socio-emotional health and development. These perceptions suggest that outdoor loose parts play will be supported and sustained at participating centres. These findings may also be of interest to researchers and practitioners exploring loose parts and active outdoor play in childcare and school settings, as understanding educator perspectives may help inform future work. It is recommended that moving forward, relationships be sought out with multidisciplinary community stakeholders to advance the understanding of the value of outdoor loose parts play, and to collectively consider how to better support children's outdoor play in early years settings. Identifying key stakeholders, and educating community members on this type of play, will help support the integration of loose parts into various spaces such as homes, child care centres, schools, and community parks. This will allow children to have quality outdoor play experiences and provide the opportunity to be physically active.

### 4.1. Strengths and limitations

The strength of this work is in its exploratory qualitative approach. While loose parts and physical activity interventions are often explored quantitatively, this project adds to the developing literature by exploring educator perspectives. Educator perspectives add to the literature as a previously underexplored area, which can help both future researchers and practitioners. Understanding what educators perceive as benefits and challenges can help future interventions be most relevant to those who will be responsible for implementing them, which may help ensure intervention sustainability. This work also adds to the literature by exploring benefits of loose parts play beyond physical activity to qualitatively understand the socio-emotional impacts of the intervention. An identified limitation is that, although the childcare centres that took part included a range of urban, rural, and socioeconomic communities, we did not specifically identify or target historically marginalized populations through this work. Future work should explore settings beyond regulated childcare, how loose parts play may be culturally relevant or appropriate in more complex settings and should explore the perspectives of traditionally underserved or excluded groups. Different play spaces may impact perceptions of loose parts play; for example, educators in childcare centres in more rural areas may have more consistent exposure to loose parts play which may result in more understanding of the benefits of this type of play on child development. Future research should seek to explore the impact of loose parts play in a wider variety of settings and populations. Future research could also explore any of the themes indicated here in more depth, or could compare loose parts play to standard playground equipment. Additionally, as this work specifically explored the perspectives of educators, future research should seek to explore the perspectives of children, families, and other key stakeholders.

## 5. Conclusions

Active outdoor play in early childhood is essential to children's health and development and is particularly valuable within childcare environments where children spend a significant proportion of their day. By providing quality outdoor play experiences and having an enriching childcare outdoor environment with loose parts, children can explore, take risks, create, and learn which together, which in turn may enhance their health and overall development. Through this study, we found educators to perceive outdoor loose parts play to have multiple social and cognitive benefits for preschool-aged children that are critical for optimal growth and development, and overall health and wellness. These favorable perceptions should support the sustainability of outdoor loose parts play within participating childcare centres. Mobilizing this knowledge to other early years advocates may further help support the integration of loose parts into various spaces in which children live, learn and play.

## References

[1] Carson V, Lee EY, Hewitt L (2017). Systematic review of the relationships between physical activity and health indicators in the early years (0–4years). BMC Public Health.

[2] LeBlanc AG, Spence JC, Carson V (2012). Systematic review of sedentary behaviour and health indicators in the early years (aged 0–4 years). Appl Physiol Nutr Metab.

[3] Poitras VJ, Gray CE, Janssen X (2017). Systematic review of the relationships between sedentary behaviour and health indicators in the early years (0–4 years). BMC Public Health.

[4] Biddle SJH, Pearson N, Ross GM (2010). Tracking of sedentary behaviours of young people: a systematic review. Prev Med.

[5] Tremblay MS, Chaput JP, Adamo KB (2017). Canadian 24-hour movement guidelines for the early years (0–4 years): An integration of physical activity, sedentary behaviour, and sleep. BMC Public Health.

[6] Tremblay MS, Carson V, Chaput JP (2016). Canadian 24-hour movement guidelines for children and youth: An integration of physical activity, sedentary behaviour, and sleep. Appl Physiol Nutr Metab.

[7] Kuzik N, Poitras VJ, Tremblay MS (2017). Systematic review of the relationships between combinations of movement behaviours and health indicators in the early years (0–4 years). BMC Public Health.

[8] Chaput JP, Colley RC, Aubert S (2017). Proportion of preschool-aged children meeting the Canadian 24-Hour Movement Guidelines and associations with adiposity: Results from the Canadian Health Measures Survey. BMC Public Health.

[9] Roberts KC, Yao X, Carson V (2017). Meeting the Canadian 24-hour movement guidelines for children and youth. Health Rep.

[10] Hesketh KR, O'Malley C, Paes VM (2017). Determinants of change in physical activity in children 0–6 years of age: A systematic review of quantitative literature. Sports Med.

[11] Lindsay AC, Greaney ML, Wallington SF (2017). A review of early influences on physical activity and sedentary behaviors of preschool-age children in high-income countries. J Spec Pediatr Nurs.

[12] Hesketh KR, Lakshman R, van Sluijs EMF (2017). Barriers and facilitators to young children's physical activity and sedentary behaviour: A systematic review and synthesis of qualitative literature. Obes Rev.

[13] Gray C, Gibbons R, Larouche R (2015). What is the relationship between outdoor time and physical activity, sedentary behaviour, and physical fitness in children? A systematic review. Int J Environ Res Public Health.

[14] Tremblay MS, Gray C, Babcock S (2015). Position statement on active outdoor play. Int J Environ Res Public Health.

[15] Statistics Canada (2019). Survey on Early Learning and Child Care Arrangements, Canada. https://www150.statcan.gc.ca/n1/daily-quotidien/190410/dq190410a-eng.htm.

[16] Bower JK, Hales DP, Tate DF (2008). The childcare environment and children's physical activity. Am J Prev Med.

[17] Wyver S (2019). The Influence of Outdoor Play on Social and Cognitive Development. Encyclopedia on Early Childhood Development. http://www.child-encyclopedia.com/sites/default/files/textes-experts/en/5223/the-influence-of-outdoor-play-on-social-and-cognitive-development.pdf.

[18] Bingham DD, Costa S, Hinkley T (2016). Physical activity during the early years: A systematic review of correlates and determinants. Am J Prev Med.

[19] Truelove S, Bruijns BA, Vanderloo LM (2018). Physical activity and sedentary time during childcare outdoor play sessions: A systematic review and meta-analysis. Prev Med.

[20] Aras S (2016). Free play in early childhood education: A phenomenological study. Early Child Dev Care.

[21] Brussoni M, Olsen LL, Pike I (2012). Risky play and children's safety: Balancing priorities for optimal child development. Int J Environ Res Public Health.

[22] Houser NE, Roach L, Stone MR (2016). Let the children play: Scoping review on the implementation and use of loose parts for promoting physical activity participation. AIMS Public Health.

[23] Johnson JE, Christie JF, Wardle F (2005). Play, development, and early education.

[24] Gibson JL, Cornell M, Gill T (2017). A systematic review of research into the impact of loose parts play on children's cognitive, social and emotional development. Sch Ment Health.

[25] Pellis SM, Pellis VC (2007). Rough-and-tumble play and the development of the social brain. Curr Dir Psychol Sci.

[26] Veitch J, Bagley S, Ball K (2006). Where do children usually play? A qualitative study of parents' perceptions of influences on children's active free-play. Health Place.

[27] Nicholson S (1971). How not to cheat children: The theory of loose parts. Landscape Archit.

[28] Änggård E (2011). Children's gendered and non-gendered play in natural spaces. Child Youth Environ.

[29] Casey T, Robertson J (2016). Loose Parts Play: A toolkit. Inspiring Scotland, Edinburgh. https://www.inspiringscotland.org.uk/wp-content/uploads/2017/03/Loose-Parts-Play-web.pdf.

[30] Flannigan C, Dietze B (2017). Children, outdoor play, and loose parts. J Child Stud.

[31] McGonigle H, Bowman-Kruhm M (2001). Think outside the sandbox: Creating natural backyard play space. Nat Life Mag.

[32] Neill P (2013). Open-Ended Materials Belong Outside Too!. High Scope.

[33] Oncu EC, Profile S, Elif A (2016). Preschoolers' usage of unstructured materials as play materials divergently. Educ J.

[34] Sutton MJ (2011). In the Hand and Mind: The intersection of loose parts and imagination in evocative settings for young children. Child Youth Environ.

[35] Gehris JS, Gooze RA, Whitaker RC (2015). Teachers' perceptions about children's movement and learning in early childhood education programmes. Child Care Health Dev.

[36] Schlembach S, Kochanowski L, Douglas BR (2018). Early childhood educators' perceptions of play and inquiry on a nature playscape. Child Youth Environ.

[37] Houser NE, Cawley J, Kolen AM (2019). A loose parts randomized controlled trial to promote active outdoor play in preschool-aged children: Physical Literacy in the Early Years (PLEY) project. Methods Protoc.

[38] Neergaard MA, Olesen F, Andersen RS (2009). Qualitative description—the poor cousin of health research?. BMC Med Res Methodol.

[39] Sandelowski M (2000). Whatever happened to qualitative description?. Res Nurs Health.

[40] Austin G, Bell T, Caperchione C (2011). Translating research to practice: using the RE-AIM framework to examine an evidence-based physical activity intervention in primary school settings. Health Promot Pract.

[41] McLeroy KR, Bibeau D, Steckler A (1988). An ecological perspective on health promotion programs. Health Educ Q.

[42] Patton MQ (2002). Qualitative Research and Evaluation Methods, 3Eds..

[43] Miles MB, Huberman AM, Huberman MA (1994). Qualitative Data Analysis: An expanded sourcebook, 2Eds..

[44] Braun V, Clarke V (2006). Using thematic analysis in psychology. Qual Res Psychol.

[45] Morrow SL (2005). Quality and trustworthiness in qualitative research in counseling psychology. J Couns Psychol.

[46] Milne J, Oberle K (2005). Enhancing rigor in qualitative description: a case study. J Wound Ostomy Continence Nurs.

[47] Nikiforidou Z (2017). ‘It is riskier': preschoolers’ reasoning of risky situations. Eur Early Child Edu Res J.

[48] Brussoni M, Gibbons R, Gray C (2015). What is the relationship between risky outdoor play and health in children? A systematic review. Int J Environ Res Public Health.

[49] Farmer VL, Williams SM, Mann JI (2017). Change of school playground environment on bullying: A randomized controlled trial. Pediatr.

