# Supplementary material for: Educator perceptions on the benefits and challenges of loose parts play in the outdoor environments of childcare centres
Source: AIMS Public Health. 2019 Oct 29;6(4):461–76. doi: 10.3934/publichealth.2019.4.461 (PMC6940583; doi:10.3934/publichealth.2019.4.461)
Supplement: Supplementary file 1 [file publichealth-06-04-461-s001.pdf]

AIMS Public Health

AIMS Public Health, 6(4): 461–476.
DOI: 10.3934/publichealth.2019.4.461
Received: 31 July 2019
Accepted: 21 October 2019
Published: 29 October 2019

http://www.aimspress.com/journal/aimsph

Research article

Educator perceptions on the benefits and challenges of loose parts play in the outdoor environments of childcare centres

Rebecca A Spencer[1,*], Nila Joshi[1], Karina Branje[1], Jessie-Lee D. McIsaac[2], Jane Cawley[3], Laurene Rehman[1], Sara FL Kirk[1,3] and Michelle Stone[1,3]

[1] School of Health & Human Performance, Dalhousie University, Halifax, Canada
[2] Faculty of Education and Department of Child and Youth Study, Mount Saint Vincent University, Halifax, Canada
[3] Healthy Populations Institute, Dalhousie University, Halifax, Canada

* **Correspondence:** Email: Becky.spencer@dal.ca; Tel: +9024941171.

Appendix A: Focus Group Questions

Focus groups followed a semi-structured guide that included the following questions:

1. Outdoor active play

- How would you describe your role as an educator in the outdoor play environment?
- What do you do when children are playing outside?
- How comfortable do you feel when children are playing outside?

2. Loose parts

- What happened when loose parts were introduced in the outdoor environment for the children? For you?
- Describe any changes you may have seen in the children's development- social, cognitive, physical, emotional, or others.

- How did your own role, attitude, and/or intentions change when loose parts were introduced into the active outdoor play environment?
- Did your usual patterns of interactions with children change? How did they change?
- Were the loose parts used equally or were there favourites? By all children?
- Which loose parts were rarely used?
- Can you describe how loose parts were used? Are some loose parts needing to be replaced or supplemented?

3. Risk-taking

- How would you define risky play?
- Describe an outdoor activity that you would consider risky for children.
- Do the children at your centre engage in risky play (provide an example)?
- Why do you think the children are engaging in this kind of play? Or why not?
- Did the introduction of loose parts add an element of risk to the children's play?
- What are your professional/personal attitudes about risk? Are they the same?

4. Policies

- What policies are in place regarding active outdoor play at your centre?
- Can you describe why these policies were implemented?
- Have these policies been affected by the PLEY project (if at all?). Will anything need to change?

5. Challenges/Benefits

- What are some of the challenges or benefits with this project?
  - ✓ With outdoor play in general?
  - ✓ With the introduction of loose parts?

6. Wrap up

- How have you grown as an educator in terms of your understanding of physical literacy and fundamental movement skills?
  - ✓ Are you more aware of PL outdoors/indoors?
  - ✓ Are you having conversations with your team and families about PL and fundamental movement skills?
  - ✓ What might be an engaging way to involve families and educators who were not involved in the PLEY Project?
- Have any of the other domains (cognitive, language, social, emotional) been enhanced following the introduction of loose parts?
  - ✓ Probe for information about changes in children's behaviours after the introduction of the loose parts into their outdoor spaces.
- How have you helped your children assess their own risk? Materials? Strategies?
- What ideas do you have for sustaining the focus on PL and FMS?

- How did the changing seasons effect the loose parts play?
- What loose parts would you like to add to your outdoor spaces? Ideas for different ones?
- What ideas do you have for storage issues?
- What is missing in our in-service and preservice training in relation to the understanding of physical literacy and fundamental movement skills?

AIMS Press

© 2019 the Author(s), licensee AIMS Press. This is an open access article distributed under the terms of the Creative Commons Attribution License (http://creativecommons.org/licenses/by/4.0)
